# Differences in the Effect of Mn^2+^ on the Reverse Osmosis Membrane Fouling Caused by Different Types of Organic Matter: Experimental and Density Functional Theory Evidence

**DOI:** 10.3390/membranes13100823

**Published:** 2023-10-05

**Authors:** Qiusheng Gao, Liang Duan, Yanyan Jia, Hengliang Zhang, Jianing Liu, Wei Yang

**Affiliations:** 1 College of Water Sciences, Beijing Normal University, Beijing 100875, China; 201931470030@mail.bnu.edu.cn (Q.G.); 201831470030@mail.bnu.edu.cn (H.Z.); 2 State Key Laboratory of Environmental Criteria and Risk Assessment, Chinese Research Academy of Environmental Sciences, Beijing 100012, China; yy_jia@tongji.edu.cn (Y.J.); liujianing20@mails.ucas.ac.cn (J.L.); 3 Institute of Water Ecology and Environment, Chinese Research Academy of Environmental Sciences, Beijing 100012, China; 4 Institute of Ecology, Chinese Research Academy of Environmental Sciences, Beijing 100012, China

**Keywords:** reverse osmosis, Mn^2+^, DFT, organic matter, membrane fouling

## Abstract

Landfill leachate from some sites contains a high concentration of Mn^2+^, which may cause reverse osmosis (RO) membrane fouling during RO treatment. In this study, the effect of Mn^2+^ on RO membrane fouling caused by typical organic pollutants (humic acid (HA), protein (BSA), and sodium alginate (SA)) was systematically investigated, and it was found that Mn^2+^ exacerbates RO membrane fouling caused by HA, SA, and HBS (mixture of HA + BSA + SA). When the Mn^2+^ concentration was 0.5 mM and 0.05 mM separately, the membrane fouling caused by HA and SA began to become significant. On the other hand, with for HBS fouling only, the water flux decreased significantly by about 21.7% and further decreased with an increasing Mn^2+^ concentration. However, Mn^2+^ has no direct effect on BSA. The effect degrees to which Mn^2+^ affected RO membrane fouling can be expressed as follows: HBS > SA > HA > BSA. The density functional theory (DFT) calculations also gave the same results. In modeling the reaction of the complexation of Mn^2+^ with the carboxyl group in these four types of organic matter, BSA has the highest energy (−55.7 kJ/mol), which predicts that BSA binding to Mn^2+^ is the most unstable compared to other organic matter. The BSA carboxylate group also has the largest bond length (2.538–2.574 Å) with Mn^2+^ and the weakest interaction force, which provides a theoretical basis for controlling RO membrane fouling exacerbated by Mn^2+^.

## 1. Introduction

Currently, landfills are still the main way to dispose of solid waste, due to their low cost [1]. However, leachate is an unavoidable problem in landfill disposal [2]. Inorganic matter, dissolved organic matter, heavy metals, and xenobiotic organic compounds are the main components of leachate, which is thought to be one of the most polluted wastewater [3]. Treating leachate is important for reducing environmental hazards and maintaining ecological balance.

Reverse osmosis (RO) is considered the most ideal method for the in situ treatment of leachate due to its high removal rate of almost any pollutant [4] and has been widely used in landfill treatment [5,6]. But, when it comes to membranes, membrane fouling is an unavoidable problem, especially under such complex water quality conditions. The high concentration and complexity of metal ions interacting with organic matter can greatly exacerbate membrane fouling and reduce RO membrane permeate flux [7].

Mitigating membrane fouling is a current research hotspot. Membrane fouling caused by the interaction between calcium, magnesium, silicon, and organic matter has been extensively studied [8,9,10]. Furthermore, membrane fouling from the interaction between aluminum and iron was reported [11,12,13,14]. However, the literature has revealed that Mn concentrations can be significant at some sites with high concentrations of organic matter [2,15], so research on the effect of Mn on RO membrane fouling caused by organic matter would be worthwhile.

There have been some studies on membrane fouling caused by Mn. As early as Kaiya et al. [16] found that membrane fouling was mainly caused by TOC and manganese when hollow fiber membranes were used in treating drinking water. Since then, some studies have also found that large quantities of manganese oxides accumulated in the long-term use of filtration membranes, resulting in severe and irreversible fouling [17,18,19,20]. However, most of the above studies about manganese fouling in membrane separation processes are on ultrafiltration, and most focus on the morphology and distribution of manganese on the membranes after long-term filtration. There are few studies on the process of manganese accumulation on RO membranes. Moreover, the effect of Mn^2+^ on RO membrane fouling caused by organic matter has not been paid enough attention.

Density functional theory (DFT) calculations have been widely used for modeling and predicting reactions between substances and have been used in membrane fouling studies [8,11,21,22]. Because of the lack of a research base related to RO membrane fouling exacerbated by Mn^2+^, DFT would be a good aid in interpreting the results. In this study, we use humic acid (HA), sodium alginate (SA), bovine serum albumin (BSA) and their mixtures (HBS, HA + BSA + SA) to simulate the typical organic matter in leachate. in the fouling experiment, the effect of Mn^2+^ was systemically studied, and combined with the DFT simulation and the experimental and DFT evidence about the work of Mn^2+^ were addressed, which can provide a theoretical basis for the control of RO membrane fouling exacerbated by Mn^2+^ and post a good case about the combination of simulation and experimentation in membrane fouling studies.

## 2. Materials and Methods

### 2.1. Chemicals and Materials

The following chemicals were used directly without further purification. BSA and HA were obtained from Sigma-Aldrich, St. Louis, MI, USA. SA manganese chloride tetrahydrate, sodium chloride, and sodium hydroxide were purchased from Shanghai McLean Biochemical Technology Co., Shanghai, China. Hydrochloric acid was purchased from Sinopharm Chemical Reagent Co., Shanghai, China. Ultrapure water (18.2 mΩcm) was obtained by Millipore Ultrapure Water Systems (Millipore, Boston, MA, USA). 

The RO membrane (BW30) was purchased from The Dow Chemical Company, Midland, TX, USA. The nylon filter membrane (0.7 μm) was purchased from Membrane Solutions, Nantong, China. 

### 2.2. Preparation of Reserve Reagent

Preparation of BSA solution: 1 g of BSA powder was dissolved in 1 L of ultrapure water and stirred for 24 h to fully dissolve it and obtain 1 g/L BSA reserve solution, which was stored at 4 °C.

Preparation of HA solution: 1 g of HA powder was dissolved in a 0.1 M NaOH so-lution, stirred continuously for 24 h, filtered with 0.7 μm membrane, adjusted the pH to neutral, and fixed to 1 L to obtain 1 g/L HA reserve solution, which was stored at 4 °C.

SA solution preparation: 1 g of SA powder was dissolved in 1 L of ultrapure water and stirred for 24 h to fully dissolve it and obtain 1 g/L SA reserve solution, which was stored at 4 °C.

Preparation of MnCl_2_ solution: 19.791 g of manganese chloride tetrahydrate was dissolved in 1 L of ultrapure water to obtain 100 mM MnCl_2_ reserve solution, which was stored at 4 °C.

### 2.3. Design of Experiments

Two series of experiments were carried out in this work (Table 1). In series 1, the organic matter concentration was set to 50 mg/L, and the Mn^2+^ concentration was set to 0, 0.01, 0.05, 0.1, 0.5, 0.8, 1, 2, 5, 8, and 10 mM, respectively.

Feed water preparation: According to the pollutant concentration setting, the organic matter reserve solution, MnCl_2_, and ultrapure water were stirred thoroughly to obtain 2.5 L of the feed solution. Finally, 1.461 g of sodium chloride solution was added to give an ionic strength of 10 mM; the pH value of the solution was between 6.22 and 6.85 without pH adjustment.

### 2.4. RO Membrane Filtration Experiment

**Figure 1:**
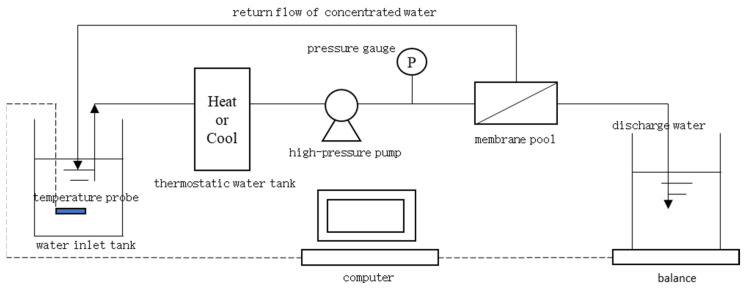
Schematic diagram of reverse osmosis experiment.

The membrane pool area is 24 cm^2^, the filtration pressure range is 1–60 bar, and the working temperature range is 0–80 °C. The principle of the high-pressure flat membrane experimental equipment is shown in Figure 1. The inlet solution enters the RO membrane pool after adjusting the temperature in the cryostat, and the high-pressure pump can provide the required pressure. Then, the water flowing out from the RO membrane pool is weighed with a balance and flows back into the inlet pool.

To ensure the accuracy of the experiments, three RO systems were used to carry out the same experiments simultaneously. The specific parameters of the RO equipment are shown in Table 1. The RO membranes were preserved in 1% NaHSO_3_ solution immersed at 4 °C, rinsed with deionized water, and soaked for 48 h before use. During the experiment, the membrane was cut into a rectangle of 4 cm × 6 cm and put into the membrane pool. when the simulated feed water was introduced, the concentrated water reflux mode was adopted, and the ultrapure water was first filtered for 12 h with a pressure of 12 bar. Then, under the premise that the flux was kept stable, the feed water was replaced with 500 mg/L NaCl solution. It continued to run for 2 h to make the membrane fully compacted, during which the desalination rate was determined using the conductivity of the feed water and the effluent. If it was greater than 85%, the membrane was deemed fully compacted. After preparing the above work, the solution in the water inlet tank is replaced with the reserve solution. After the preliminary experimental simulation, there was a significant decline in water flux after 6 h of operation, so the experimental membrane filtration time in this work was set to 6 h.

The membrane permeate flux (J, m^3^/(m·s^2^)) was calculated as follows (Equation (1)):

(1) $$J=\frac{\Delta m/(60\times 10)}{\rho \cdot A\cdot 100}$$

where A is the effective membrane area (cm^2^), ρ is the density of produced water (g/cm^3^), and Δm is the product water quality per 10 min (g/min).

The initial flux at the starting phase of the fouling period (5 min) is known as J_0_. J/J_0_, on behalf of the flux ratio at any moment during the experiment, was identified as the normalized flux [9].

### 2.5. RO Membrane Characterization

The plugged RO membrane was removed from the flat membrane module, cut into 0.5 cm × 0.5 cm sizes, and placed at room temperature to dry for at least 24 h at the end of the membrane plugging experiment. The membrane surface was characterized using SEM/EDS.

### 2.6. The Density Functional Theory (DFT) Calculations

The density functional theory (DFT) calculations were carried out with the VASP code [23]. The Perdew–Burke–Ernzerhof (PBE) function of the generalized gradient approximation (GGA) [24] was used to process the exchange–correlation. The projector augmented-wave pseudopotential (PAW) [25] was applied with a kinetic energy cut-off of 500 eV, which was utilized to describe the expansion of the electronic eigenfunctions. The vacuum thickness was set to 25 Å to minimize interlayer interactions. The Brillouin zone integration was sampled using a Γ-centered 5 × 5 × 1 Monkhorst–Pack k-point. All atomic positions were fully relaxed until the energy and force reached a tolerance of 1 × 10^−5^ eV and 0.03 eV/Å, respectively. The dispersion-corrected DFT-D method was employed to consider the long-range interactions [26].

The binding energy (Ebind) of a complex formed between two molecules, A and B, can be calculated using the following equation (Equation (2)):

E_bind_ = E_complex_ − (E_A_ + E_B_) (2)

where E_complex_ is the total energy of the molecular complex of A and B; E_A_ and E_B_ are the total energies of isolated molecules A and B, respectively.

## 3. Results and Discussion

### 3.1. Effect of Mn^2+^ on RO Membrane Fouling Caused by HA

**Figure 2:**
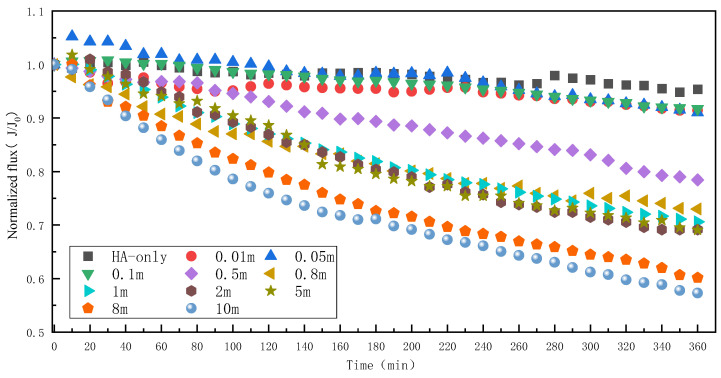
Effect of different Mn^2+^ concentrations on RO membrane fouling caused by HA.

The change in water flux is the most direct indicator of membrane fouling. The effect of different Mn^2+^ concentrations on the HA fouling when the HA concentration is 50 mg/L is shown in Figure 2. When there is only HA in the feed water, the water flux of the RO membrane only decreases by 4.7% after 6 h of operation, which indicates that HA alone does not cause significant fouling of the RO membrane. Li et al. [7], in their study of RO membrane fouling with HA and silicon, found no significant change in water flux when the HA concentration was 50 mg/L, consistent with this study. It has also been found that the water flux remained insignificant at HA concentrations of 10–90 mg/L [27]. With the increase in the Mn^2+^ concentration, the water flux began to decrease significantly. When the Mn^2+^ concentration was 0.01, 0.05, and 0.1 mM, the decrease in the water flux was small, only 8.3–9.0%. But when the concentration increased to 0.5 mM, the water flux showed a notable decrease of 21.6%. Finally, when the Mn^2+^ concentration was 10 mM, the water flux decreased by 42.7%. Overall, HA itself had no significant effect on RO membrane fouling. However, the addition of Mn^2+^ exacerbated RO membrane HA fouling and increased the degree of membrane fouling as the concentration of Mn^2+^ increased, producing a synergistic contamination effect between the two pollutants, which is similar to that of the currently known roles of Ca^2+^ and Mg^2+^ in the HA fouling [28,29]. 

**Figure 3:**
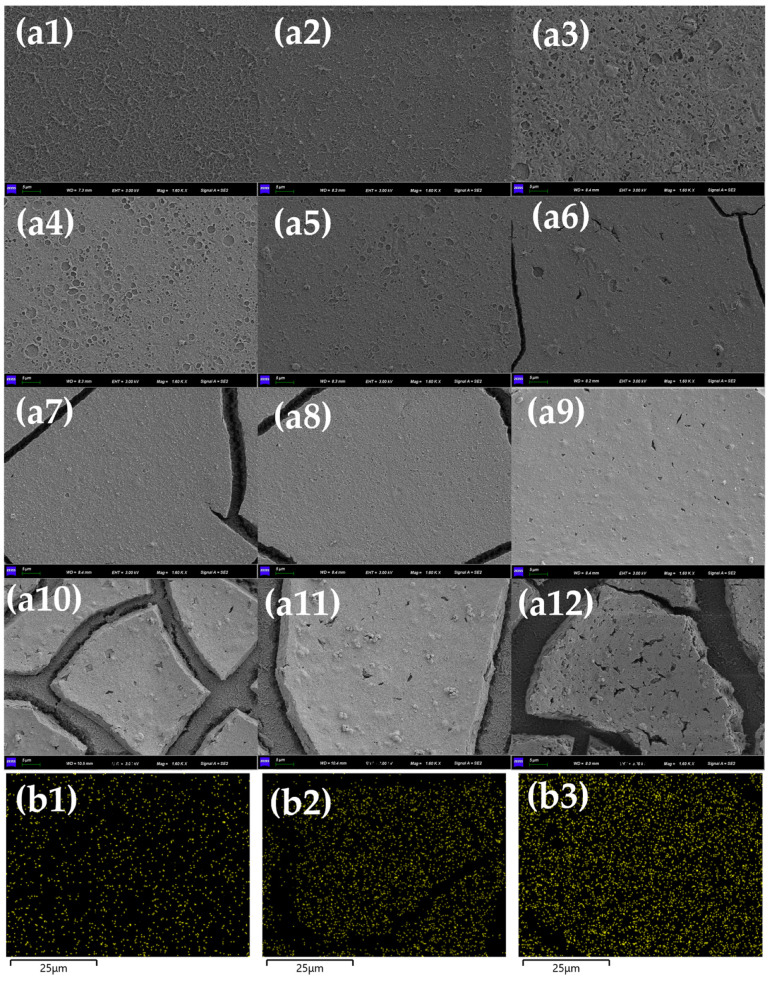
(**a**) Morphology of RO membrane, concentration of HA = 50 mg/L, (**a1**–**a12**): no-fouled RO membrane, HA + 0 mM Mn^2+^, HA + 0.01 mM Mn^2+^, HA + 0.05 mM Mn^2+^, HA + 0.1 mM Mn^2+^, HA + 0.5 mM Mn^2+^, HA + 0.8 mM Mn^2+^, HA + 1 mM Mn^2+^, HA + 2 mM Mn^2+^, HA + 5 mM Mn^2+^, HA + 8 mM Mn^2+^, and HA + 10 mM Mn^2+^; (**b**) EDS mapping of RO membrane, concentration of HA = 50 mg/L, (**b1**–**b3**) HA + 0.05 mM Mn^2+^, HA + 1 mM Mn^2+^, and HA + 10 mM Mn^2+^.

To further investigate the effect pattern of Mn^2+^ on the HA fouling of the RO membrane, the morphology of the RO membrane was examined using SEM, while the HA concentration was 50 mg/L (Figure 3). The surface of the unfouled RO membrane was rougher with obvious fiber structure and dense voids, and the membrane fouling was only caused by HA, which had a reduced fiber structure and roughness compared to the pristine membrane but still had more voids [30]. With the increase in Mn^2+^ concentration, the surface of the RO membrane was gradually covered by Mn^2+^-HA fouling, and the membrane morphology also showed a large difference. Even if the Mn^2+^ concentration is 0.01 mM, a layer of fouling can be seen on the membrane. When the Mn^2+^ concentration increased to 0.05 mM and 0.1 mM, the membrane surface roughness was further reduced but still retained some pore structure. However, until the Mn^2+^ concentration increased to 0.5 mM, the thickness of the fouling on the membrane increased significantly, and the void structure completely disappeared; meanwhile, the membrane fouling significantly increased, which is consistent with the change in water flux. With the further increase in Mn^2+^ concentration, the fouling accumulated on the membrane was thicker and denser. In addition, with the increase in Mn^2+^ concentration, more severe Mn^2+^ fouling on the membrane surface was also present in the corresponding EDS patterns.

### 3.2. Effect of Mn^2+^ on RO Membrane Fouling Caused by SA

**Figure 4:**
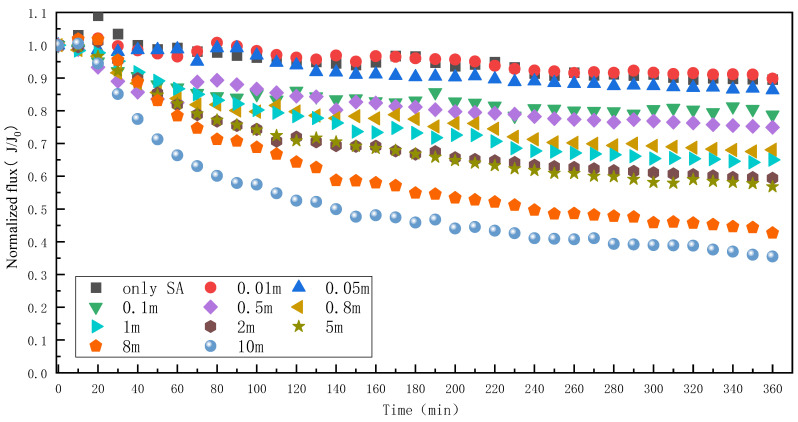
Effect of different Mn^2+^ concentrations on RO membrane fouling caused by SA.

When the SA concentration was 50 mg/L, the effect of different Mn^2+^ concentrations on the SA fouling of the RO membrane was as shown in Figure 4. When only SA was in the feed water, the water flux decreased by about 10.6% after 6 h of operation, and the degree of fouling was higher than that of HA, mainly because SA could form a three-dimensional reticulation colloid [31], which resulted in more serious membrane fouling. Unlike HA, a significant decrease in water flux was observed when the Mn^2+^ concentration was only 0.05 mM. With the increase in Mn^2+^ concentration, the water flux further dropped until the water flux decreased by 64.5% when the Mn^2+^ concentration reached 10 mM. The interaction between Mn^2+^ and SA molecules is stronger than HA, and the presence of Mn^2+^ greatly enhances the strength of SA molecules binding to each other, exacerbating membrane fouling. Mn^2+^ may play a bridging role in SA, bridging the two-dimensional SA structure into a larger structure of three-dimensional pollutants and accumulating in the membrane.

**Figure 5:**
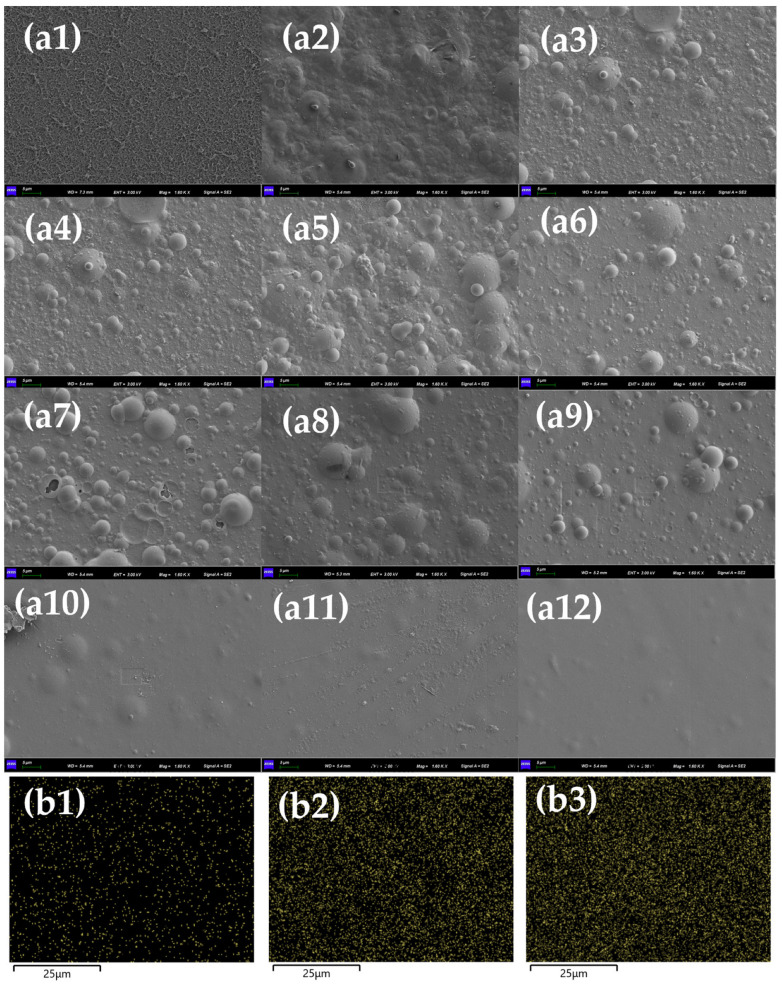
(**a**) Morphology of RO membrane, concentration of SA = 50 mg/L, (**a1**–**a12**) unfouled RO membrane, SA + 0 mM Mn^2+^, SA + 0.01 mM Mn^2+^, SA + 0.05 mM Mn^2+^ SA + 0.1 mM Mn^2+^, SA + 0.5 mM Mn^2+^, SA + 0.8 mM Mn^2+^, SA + 1 mM Mn^2+^, SA + 2 mM Mn^2+^, SA + 5 mM Mn^2+^, SA + 8 mM Mn^2+^, and SA + 10 mM Mn^2+^; (**b**) EDS mapping of RO membrane, concentration of SA = 50 mg/L, (**b1**–**b3**) SA + 0.05 mM, Mn^2+^ SA + 1 mM, Mn^2+^, and SA + 10 mM Mn^2+^.

When the concentration of SA was 50 mg/L, the effect of Mn^2+^ concentration on RO membrane morphology is shown in Figure 5. Compared with the unfouled pristine RO membrane, the membrane fouling caused by SA alone was visibly clogged, with a small number of spherical projections on the membrane surface. As the Mn^2+^ concentration increased to 0.8 mM, the density of spherical projections increased, but the roughness of the membrane surface decreased. However, when the Mn^2+^ concentration increased from 1 mM to 10 mM, the spherical projections gradually disappeared, and a dense and smooth fouling layer was formed on the membrane surface, which led to a rapid decrease in water flux. The EDS mapping of Mn was further analyzed, and similar to the HA membrane fouling, the intensification of the SA membrane fouling was directly proportional to the distribution density of elemental Mn, which further proved the important role of Mn^2+^ in membrane fouling.

### 3.3. Effect of Mn^2+^ on RO Membrane Fouling by Caused BSA

**Figure 6:**
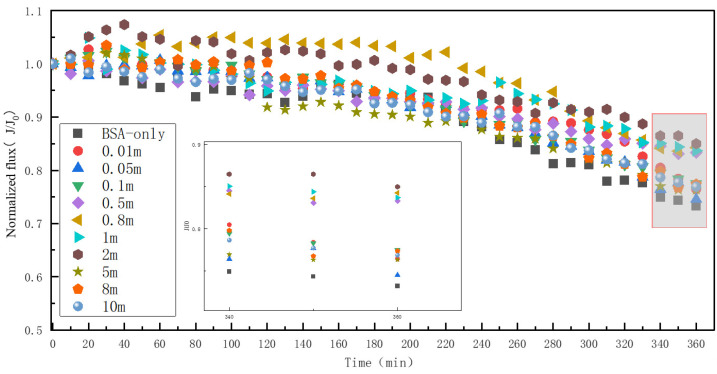
Effect of different Mn^2+^ concentrations on RO membrane fouling caused by BSA.

The effect of different concentrations of Mn^2+^ on BSA fouling of RO membranes at a BSA concentration of 50 mg/L is shown in Figure 6. Different from HA and SA, the water flux decreased by 26.8% when there was only 50 mg/L of BSA within the feed water, and the membrane fouling was the most serious. As the concentration of Mn^2+^ increased, the membrane fouling showed a slow recovery until the concentration of 2 mM, and the water flux recovered by 11%. But, when the concentration continued to increase to 5 mM, the water flux decreased by about 8.6%, remained stable and did not change with the increase in the concentration of Mn^2+^. It is possible that when only BSA was present, the BSA molecules aggregated with each other through the cross-linking disulfide bonds [32], causing membrane fouling.

**Figure 7:**
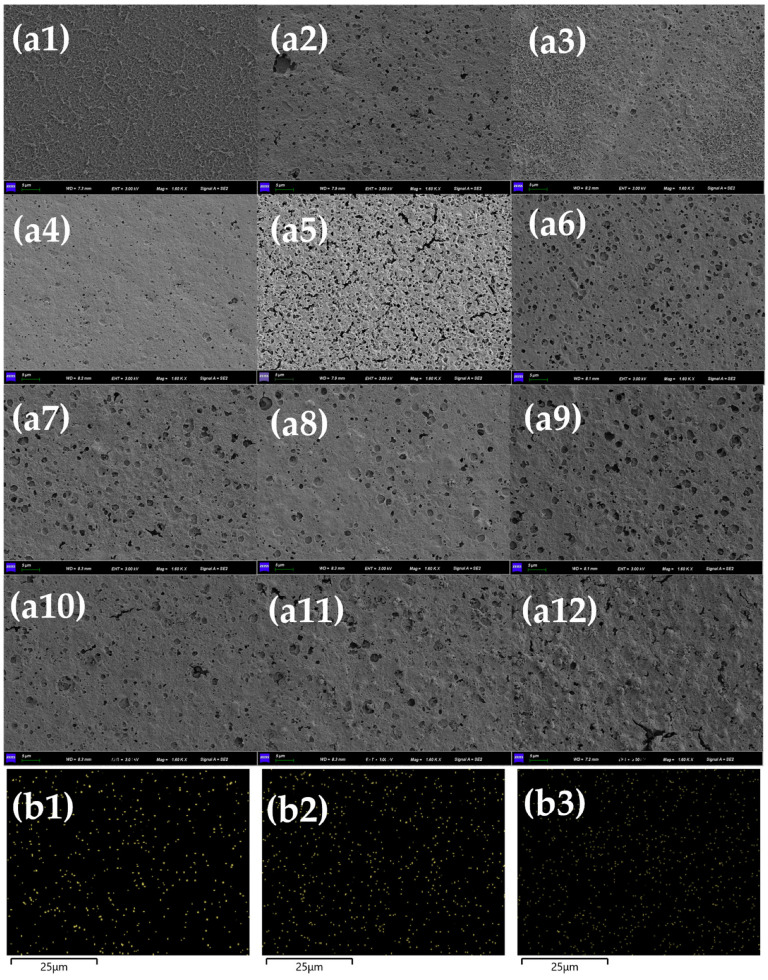
(**a**) Morphology of RO membrane, concentration of BSA = 50 mg/L, (**a1**–**a12**) unfouled RO membrane, BSA + 0 mM Mn^2+^, BSA + 0.01 mM Mn^2+^, BSA + 0.05 mM Mn^2+^, BSA + 0.1 mM Mn^2+^, BSA + 0.5 mM Mn^2+^, BSA + 0.8 mM Mn^2+^, BSA + 1 mM Mn^2+^, BSA + 2 mM Mn^2+^, BSA + 5 mM Mn^2+^, BSA + 8 mM Mn^2+^, and BSA + 10 mM Mn^2+^; (**b**) EDS mapping of RO membrane, concentration of BSA = 50 mg/L, (**b1**–**b3**) BSA + 0.05 mM Mn^2+^, BSA + 1 mM Mn^2+^, and BSA + 10 mM Mn^2+^.

Figure 7a shows the RO morphology under BSA fouling. After filtering 50 mg/L BSA solution, the membrane was covered with a porous BSA membrane, and although the water channels on the RO membrane were covered by the BSA pollutant, its porous characteristics still ensured a sizable water flux (73.2%). With the increase in Mn^2+^ concentration to 2 mM, the pores on the surface of the fouled membrane gradually increase, explaining the phenomenon of water flux increase. Afterward, the Mn^2+^ concentration further increased to 10 mM, the pores on the membrane gradually decreased, the membrane thickness increased, and the water flux decreased, though it was still higher than the case of 50 mg/L BSA fouling alone. The results of the EDS mapping analysis of Mn elements on the membrane surface are shown in Figure 6b, and the distribution density of Mn elements has nothing to do with the membrane fouling and the Mn^2+^ concentration in the feed water; there is no interaction between Mn^2+^ and BSA.

This difference in BSA fouling may be because Mn^2+^ exacerbates the salting-out phenomenon. Mo et al. [33] found a similar phenomenon for Mg^2+^, which, because of its high charge density, causes a more significant salting-out effect. When BSA alone is present, membrane fouling is caused by the mutual aggregation of BSA molecules through the cross-linking disulfide bonds when membrane fouling is the most serious. On the other hand, with the addition of Mn^2+^, BSA is no longer aggregated, but a salting-out effect occurs, resulting in the deposition of individual BSA molecules on the membrane; the fouling layer remains porous, although the fouling layer is thickened.

### 3.4. Effect of Mn^2+^ on RO Membrane Fouling Caused by HBS

**Figure 8:**
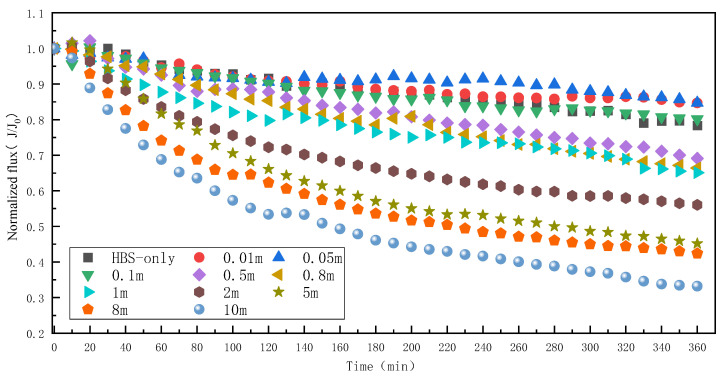
Effect of different Mn^2+^ concentrations on RO membrane fouling caused by HBS.

When the concentration of HBS was 50 mg/L (20 mg/L HA + 15 mg/L BSA + 15 mg/L SA), the effects of different concentrations of Mn^2+^ on RO membrane fouling were as shown in Figure 8. Compared with HA, SA, and BSA, the membrane fouling caused by HBS combines the characteristics of the three pollutants. When only HBS was present in the RO system, the water flux decreased by about 21.7%, similar to that in the presence of only BSA. Existing studies have found that when HA and BSA are present in the system, the two organisms will aggregate regardless of the presence of multivalent ions [22], and at this time the decrease in water flux may be due to the interaction of HA-BSA or the action of BSA alone. However, when the Mn^2+^ concentration in the system increased from 0 to 0.1 mM, the water flux gradually recovered, similar to the fouling characteristics of BSA in the previous text. This may be due to the limited interaction between organic matter and Mn^2+^ at a low Mn^2+^ concentration, and the membrane fouling is mainly dominated by BSA. When the Mn^2+^ concentration increased to 0.5 mM, there was a significant decrease in water flux, and this critical concentration was consistent with that of HA fouling. The water flux decreased rapidly with the increase in Mn^2+^ concentration, and this decrease rate was characterized by SA fouling. When the Mn^2+^ concentration reached 10 mM, the water flux decreased by 66.8%, which is the maximum decrease value in this work. The above results showed that Mn^2+^ exacerbated the membrane fouling caused by HBS, and the degree of membrane fouling caused by the mixture of the three organics was much higher than those caused by any organics.

**Figure 9:**
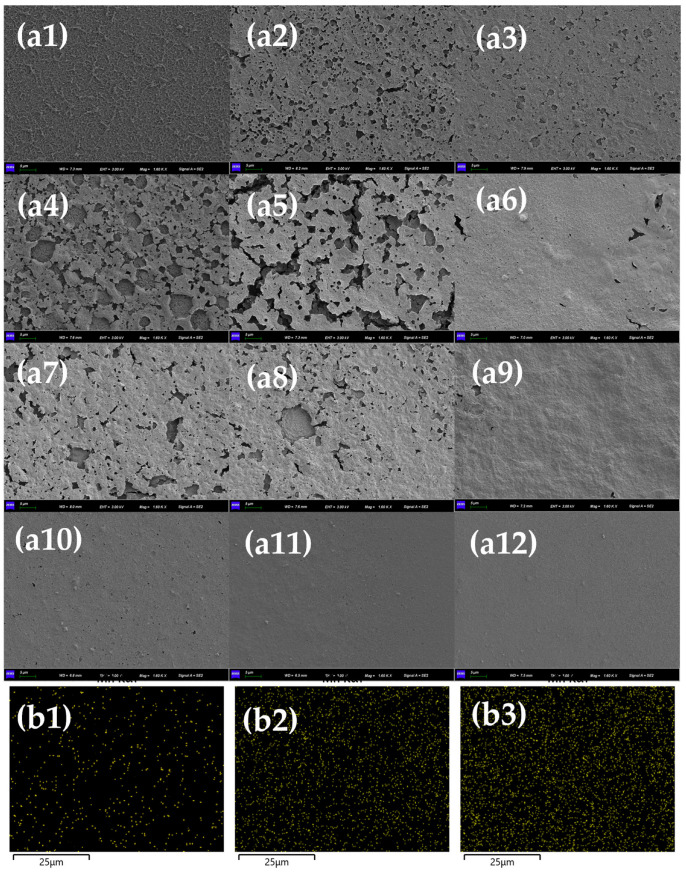
(**a**) Morphology of RO membrane, concentration of HBS = 50 mg/L, (**a1**–**a12**) unfouled RO membrane, HBS + 0 mM Mn^2+^, HBS + 0.01 mM Mn^2+^, HBS + 0.05 mM Mn^2+^, HBS + 0.1 mM Mn^2+^, HBS + 0.5 mM Mn^2+^, HBS + 0.8 mM Mn^2+^, HBS + 1 mM Mn^2+^, HBS + 2 mM Mn^2+^, HBS + 5 mM Mn^2+^, HBS + 8 mM Mn^2+^, and HBS + 10 mM Mn^2+^; (**b**) EDS mapping of RO membrane, concentration of HBS = 50 mg/L, (**b1**–**b3**) HBS + 0.05 mM Mn^2+^, HBS + 1 mM Mn^2+^, and HBS + 10 mM Mn^2+^.

The SEM image of the RO membrane is shown in Figure 9a. The surface of the membrane foul with 50 mg/L HBS was covered by a layer of fluffy and porous fouling, which was similar to the morphology of the membrane fouling caused by BSA alone. With the increase in Mn^2+^ concentration from 0 mM to 0.1 mM, the pore space of the fouled layer on the membrane increased and became fluffier, which was characterized by obvious BSA fouling and was consistent with the change rule of water flux, indicating that the fouling of the HBS membrane was dominated by BSA when the Mn^2+^ concentration level was low. With the further increase in Mn^2+^ concentration, the fouled layer became smooth and dense, with the morphological characteristics of HA and SA membrane fouling at the same time, and the change rule of water flux remained consistent. At the same time, the EDS mapping (Figure 8b) of elemental Mn also showed that the distribution density of elemental Mn on the RO membrane increased with the increase in membrane fouling. It further illustrated that the presence of Mn^2+^ exacerbated the HBS fouling of the RO membrane.

### 3.5. Validation Using DFT

The effect of Mn^2+^ on RO membrane fouling caused by different organic matter varies widely. Influenced by manganese ions, the mixture of SA, HA, and BSA caused the most significant RO membrane fouling, followed by SA and HA, whereas BSA appeared to be unaffected by manganese ions, with the main fouling coming from BSA itself. This is quite different from the role of the Ca^2+^ and Mg^2+^. Without having to do additional characterization experiments, DFT calculations are a great tool that can be used to validate and interpret this result. 

In the realm of Mn^2+^ interactions with organic compounds, the ability of Mn^2+^ to engage in multiple coordination bonds with various oxygen-containing functional groups within these organic molecules plays a pivotal role. Among these functional groups, carboxyl groups, often carrying a negative charge, exhibit a notably heightened affinity for manganese ions. This discrepancy in binding strength stems from the differences in the chemical structure and functionalities of the organic molecules, which, in turn, influence the intensity of their interactions with Mn^2+^.

Coordination bonds, the focal point of this interplay, refer to the interactions between Mn^2+^ and one or more ligands, typically atoms or molecules with unpaired electrons, facilitated by sharing electron pairs to form chemical bonds. Mn^2+^ can form multiple coordination bonds due to possessing numerous unpaired electron pairs.

When Mn^2+^ associates with organic compounds, they can establish coordination bonds with oxygen-containing functional groups present in these substances. These functional groups encompass carboxyl (-COOH) and hydroxyl (-OH), oxygen atoms in sugar rings, and glycosidic bonds. The interaction between Mn^2+^ and these groups is achieved by sharing electron pairs. Notably, carboxyl groups bearing negative charges emerge as particularly effective functional groups in this context due to the presence of electron-rich oxygen atoms within their carboxyl groups. That is why carboxyl groups tend to bind more readily to manganese ions than other functional groups.

**Figure 10:**
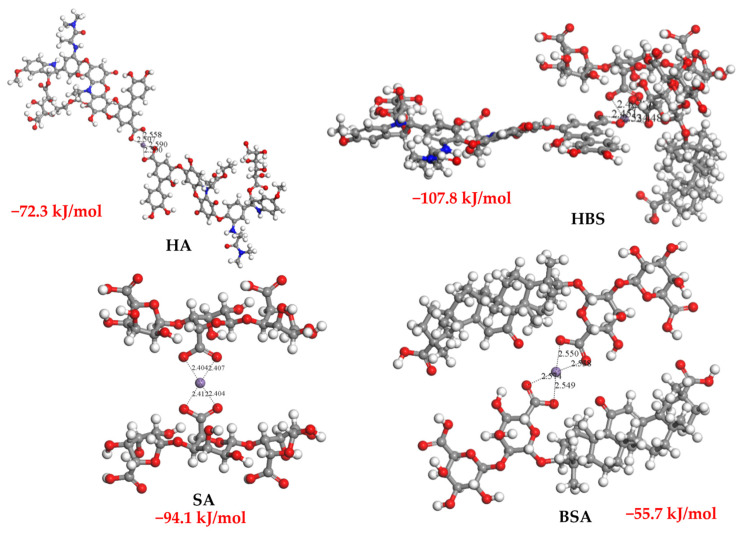
Structure and energies in the stable state of Mn^2+^ binding to various organic pollutants.

The capacity of Mn^2+^ to form complexes with -COOH of different types of organic matter obtained via DFT calculations (Figure 10) agrees with the experimental results; thus, the order is HBS > SA > HA > BSA. BAS has the highest energy (−55.7 kJ/mol) for stabilizing structures, which predicts that BSA binding to Mn^2+^ is the most unstable compared to the other three organics. The BSA carboxylate group also has the largest bond length (2.538–2.574 Å) with Mn^2+^, showing the weakest interaction force. As a result, BSA and Mn^2+^ bind to a limited extent.

## 4. Conclusions

The presence of Mn^2+^ enhanced the aggregation of HA, SA, and HBS, thereby leading to deposition on the membrane surface and causing more severe membrane fouling. When the Mn^2+^ concentration was 0.5 mM and 0.05 mM separately, the membrane fouling caused by HA and SA began to become significant, while with HBS fouling only, the water flux decreased significantly by about 21.7%, and decreased further with increasing Mn^2+^ concentrations. However, Mn^2+^ has no direct effect on BSA. The degrees to which Mn^2+^ affected membrane fouling were in the order HBS > SA > HA > BSA, which was confirmed via DFT calculations. In modeling the reaction of the complexation of Mn with these three types of organics, BSA has the highest energy (−55.7 kJ/mol), which predicts that BSA binding to Mn^2+^ is the most unstable; compared to the other three organics, the BSA carboxylate group also has the largest bond length (2.538–2.574 Å) with Mn^2+^, showing the weakest interaction force. This study provides a theoretical basis for the control of RO membrane fouling exacerbated by Mn^2+^.

This study explores the feasibility of using DFT calculations to interpret the experimental results. Although better results were obtained, the DFT results can only be used as a reference; the results still need to be analyzed in depth in conjunction with other data in the next study.

## Figures and Tables

**Table 1:** Operating parameters of RO test equipment.

Item	Parameter
Membrane Size	24 cm^2^
Operation method	Cross-flow filtration + concentrate recirculation
RO Membrane material	Polyamide composite membrane
Operating pressure	12 bar
Filtration time	6–12 h
Crossflow rate	about 9.6 cm/s
Working voltage	220 V
Working temperature	25 °C

## Data Availability

The data presented in this study are available on request from the corresponding author.
